# Tier 3 specialist weight management service and pre‐bariatric multicomponent weight management programmes for adults with obesity living in the UK: A systematic review

**DOI:** 10.1002/edm2.42

**Published:** 2018-10-25

**Authors:** Mohammed Alkharaiji, Uchenna Anyanwagu, Richard Donnelly, Iskandar Idris

**Affiliations:** ^1^ Department of Surgery, Graduate Entry Medical School Royal Derby Hospital, University of Nottingham Derby UK; ^2^ Faculty of Public Health, College of Health The Saudi Electronic University Riyadh Saudi Arabia

**Keywords:** bariatric, systematic review, Tier 3, weight management

## Abstract

**Background:**

NHS England has recommended a multidisciplinary weight management services (MWMS—Tier 3 services) for patients requiring specialized management of obesity, including bariatric surgery, but clinical and measurable health‐related outcomes from these services remains fragmented. We therefore undertook a systematic review to explore the evidence base of effect on body weight loss and comorbidities outcomes of Tier 3 or UK pre‐bariatric MWMPs.

**Methods:**

AMED, CINAHL, EMBASE, HMIC, MEDLINE, PsycINFO, PubMed, HDAS search and Google Scholar were searched from January 2000 to September 2017 in a free‐text fashion and crossed‐references of included studies to identify potential illegibility. Inclusion criteria were as follows: (a) published Tier 3 original study abstracts/articles; (b) intervention studies with before and after data; (c) studies that included any sort of MWMPs conducted on British residents with obesity; and (d) studies included T2DM measurements in a MWMPs.

**Results:**

In total, 19 studies met the inclusion criteria. The total number of participants analysed was N = 11,735. Baseline accumulative average BMI was calculated at 42.54 kg/m^2^, weight 117.88 kg and waist circumference 126.9 cm. And at 6 months, 40.73 kg/m^2^, 112.17 kg and 120.3 cm, respectively. Secondary outcome variables were as improved with reduction in HbA1c, fasting blood sugars, insulin usage and blood pressure. Physical activity increased at 3 months then declined after 6 months with no significant changes in cholesterol levels.

**Conclusion:**

Tier 3 and MWMPs have a short to mid‐ranged positive effect on obese patients (BMI ≥30 kg/m^2^) living in the UK regarding accumulated reduction in weight, glycaemic control, blood pressure and with subtle improvements in physical activity.

## 1 BACKGROUND

Morbid obesity is an increasing lifelong chronic condition that no country has yet succeeded to tackle.^1^ In England, the prevalence of obesity is among the highest in Europe.^2^ Two‐thirds of adults are overweight and one in four are obese (Body Mass Index (BMI) of >30 kg/m^2^).^3^, ^4^ McKinsey Global Institute reported that, second to smoking, obesity has the largest impact on the public health budget with an estimated annual cost to the United Kingdom's (UK) National Health Service (NHS) of £44.7b.^5^ The importance of a range of obesity prevention initiatives comes from the increasing number of health complications and their related high cost. High Blood Pressure (BP), type 2 diabetes mellitus (T2DM), heart attacks, strokes, cancers and other health issues, for instance, are evidently associated to the conditions of being overweight or obese.^4^

Even though bariatric surgical intervention is a proven effective approach for treating chronic obesity, access and eligibility for bariatric surgery remains low.^6^ The reasons for this are multifactorial, but may include a lack of developed infrastructure for medical assessment and services, unclear referral procedures, as well as uncertainties regarding costs and long‐term outcomes.^7^ In England, the rate of bariatric surgical operations dropped by 31% between 2011‐2012 and 2014‐2015 (from 8794 to 6032 operations, respectively).^4^ It is much worse in Scotland and Wales, and there is no NHS bariatric surgery performed in Northern Ireland.^8^ Provision of bariatric intervention by NHS is, therefore, less than 1% of the national need.^8^

In the UK, obesity is managed through a 4‐levels tiered pathway. Tier 1 and 2 are focused on universally environmental and population‐wide prevention services.^4^, ^9^ Following this, individuals with more complex obesity and/or medical needs are considered for Tier 3 Multidisciplinary Weight Management Service (MWMS),^10^ which may lead to a Tier 4 service for consideration of bariatric surgery.^4^, ^11^ Tier 3 MWMS consists of a (bariatric) physician, a dietitian, a specialist nurse and a clinical psychologist with access to physical therapy.^4^ All adults identified with a BMI of ≥40 kg/m^2^, or ≥35 kg/m^2^ with comorbidities are eligible for bariatric surgery following assessment and input from Tier 3 services. Tier 3, in this context, could also apply to a “Weight Assessment and Management Clinic” provided by primary or secondary care.^4^

Within a Tier 3 service, strategies are implemented to make critical changes about eating and physical activity habits to improve health and identify risk factors so that the planned intervention addresses and improves all elements comprehensively.^4^ Screening for hormonal or genetic causes of excessive weight as well as all related comorbidities and disabilities are conducted by the bariatric physician and each individual should have their own tailored lifestyle and healthful eating advice provided by a specialist dietitian. In addition, patients are screened for signs of psychiatric comorbidities due to the well‐recognized link between obesity with many psychological disorders such as anxiety, depression, self‐harm and suicidal behaviours, eating disorders (such as binge eating and bulimia nervosa), borderline personality disorders, alcohol and substance misuse, childhood adversity, among others. Patients with proven effort, an adequate timeframe prescribed by the multidisciplinary team, and with right weight criteria and medically optimized for surgery, will then be advised to progress towards the Tier 4 bariatric surgical intervention.^4^, ^12^

Although our understanding of the benefits of a Tier 3 service is growing—based on our appraisal of current literature,^11^, ^13^ current evidence remains fragmented and needs to be synthesized to produce a more comprehensive picture which will help to translate to a safe and cost‐effective approach to the management of morbid obesity in the UK. We, therefore plan to explore the evidence base of effect magnitude on body weight loss in addition to other health‐related outcomes of severely obese adults undergoing a Tier 3 or pre‐bariatric Multicomponent Weight Management Programmes (MWMPs) in the UK. We include obese adults in the UK with a BMI ≥30 kg/m^2^ who have been enrolled in a Tier 3 service or in any form of MWMP for losing weight.

## 2 METHODS

### 2.1 Literature search

**Figure 1:**
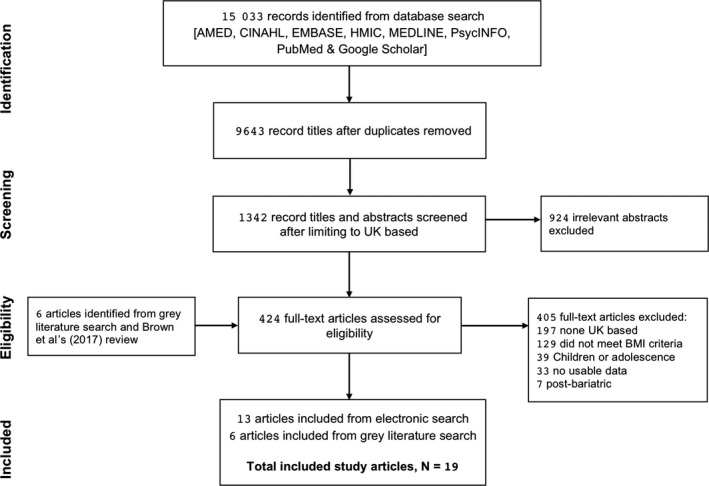
Preferred reporting items for systematic reviews and meta‐analyses (PRISMA) flow chart^37^

A free‐text literature search of articles published from January 2000 through September 2017 was performed. The search used the Healthcare Databases Advances Search (HDAS) via the National Institute for Health and Care Excellence's (NICE) evidence services with access to the following electronic bibliographical databases: AMED, CINAHL, EMBASE, HMIC, MEDLINE, PsycINFO and PubMed. An extended search was conducted using Google Scholar after reviewing additional studies that were included by Brown et al (2017) systematic review.^12^ Terms used were related to “obesity” and “overweight” in conjunction with geographical restrictions to the UK (eg, England, Wales, Scotland, North Ireland). Terms related to MWMS, Specialist Weight Management (SWM) and Tier 3 (eg, weight management services, weight reduction programmes, weight management interventions, multidisciplinary weight loss initiatives and multicomponent weight loss schemes) were utilized on the titles and abstracts search. In addition, we screened reference sections of all included studies to identify potential illegible articles that meet the inclusion criteria of this review. See Figure 1 flow chart.

### 2.2 Study selection

In this review, we use a similar pragmatic selection approach to Brown et al (2017).^12^ Tier 3 studies for adults (18 years and over with no upper age limit) with a mean baseline BMI of ≥40 or ≥35 kg/m^2^ with a comorbidity or ≥30 kg/m^2^ with T2DM are included. In addition, all UK multicomponent pre‐bariatric weight loss interventions that were planned and delivered for obese adults with BMI ≥30 kg/m^2^ published since January 2000 until September 2017 were screened for potential inclusion. Inclusion criteria follow: (a) published Tier 3 original study abstracts and articles; (b) intervention studies with before and after data; (c) studies including any sort of MWMP planned for morbidly obese British residents; and (d) studies that included T2DM measurements in a MWMP for overweight adults. We excluded studies on children or adolescents and all studies conducted within non‐British weight reduction intervention programmes. The decision to include or exclude studies was initially made based on the article title, then abstract and finally review the full‐text article.

### 2.3 Data extraction

We evaluated each of the included studies and extracted four data aspects: (a) descriptive to study design and intervention (Table S1); (b) sample size and demographic characteristics (Table S2); (c) assessed measurements (Table S3); and (d) health outcome records at baseline followed by points of time intervals (Tables S4‐S9). For each segment, authors (year and country where intervention was delivered) are indicated.

In the descriptive of study design and intervention, we included the following: sitting, study design, aim, type of intervention, a brief description of intervention, inclusion and exclusion criteria, duration and lost‐to‐follow‐up or drop‐out data rate. In the demographics: sample size (N), age (years), gender (female, %), ethnicity, socioeconomic status (SES), education level, marital status and type of financial support. On the assessed measurements (n, %): mental disorder, anxiety, depression, sleep apnoea, hypertension, cardiovascular disease (CVD), ischaemic heart disease, hyperlipidaemia, diabetes mellitus (DM), impaired fasting glucose, insulin use, oral hypoglycaemic and incretin based.

For the baseline, 3, 6,12, 18 and 24 months, we extracted (or calculated) the following variables of health outcome results: BMI (kg/m^2^), weight (kg), waist circumference (cm), 5% or more weight loss achieved (per cent), 10% weight loss achieved (per cent), lost ≥5 kg (per cent), lost ≥10 kg (per cent), lost 0 to ≤5 kg (per cent), lost 5 to ≤10 kg (per cent), lost 10 to ≤15 kg (per cent), lost 15 to ≤20 kg (per cent), lost ≥20 kg (per cent), mean weight loss (kg and Standard Deviation [SD]), percentage of body weight lost, BP (systolic and diastolic), hypertension, insulin usage, Fasting Blood Sugar (FBS) (mmol/L), glucose (mmol/L), HbA1c^1^ (% and mmol/mol^‐1^), cholesterol (mmol L^‐1^), HDL and LDL (mmol L^‐1^), total cholesterol, triacylglycerol and levels of physical activity.

We were not able to extract food intake records because of heterogeneity of stratification methods used by a number of studies in addition to concerns of related recall bias. We support Brown et al’s (2017) decision regarding the difficulty in producing a meta‐analysis in reviewing Tier 3 and all MWMPs due to heterogeneity.^12^ The increased rate of patient drop‐out and apparent risk of bias are also preventive factors to a meta‐analysis. Thus, narrative synthesis is carried out.

### 2.4 Risk of bias assessment

Two authors (MA and UA) have independently assessed all included studies using the Cochrane Handbook for Systematic Reviews of Intervention tool.^14^ They evaluated the possibility of the following bias elements: allocation sequence, allocation concealment, blinding (of participants, personnel and outcome assessors), incomplete outcome data and selective outcome for reporting or publication of data.

## 3 RESULTS

1,342 article abstracts were identified as potentially relevant, and after reviewing 418 in full‐text, 11 articles and 2 published study abstracts met the inclusion criteria and were included in the review. Grey literature search and reference lists check including Brown et al’s (2017) systematic review yielded additional 6 study articles (see Figure 1 Flow chart). In total, 19 studies are eligible for inclusion. The reasons for excluding 405 articles were as follows: a) not being a UK intervention; b) not Tier 3 or MWMP; c) did not meet BMI criteria; d) intervention intended for children or adolescents; e) no usable data (eg, qualitative approach for satisfactory records); and f) post‐bariatric groups comparison. The 19 included studies were all published within the last 17 years in 15 different journals, all conducted in the UK.

Table 1 present study design as well as an intervention type and descriptive summary of all included studies, demographic characteristics of participants (N = 11,735), reported measurements and baseline characteristics and average reported health outcome results in three, six, twelve, eighteen, twenty‐four‐month intervals. The clear majority of studies (90%) did not reach 18 and 24 months, thus reporting MWMS true effect at these particular points of time was difficult. However, a decision was made to evaluate findings at the last endpoint possible as this may add value.

**Table 1:** List of included studies with summary characteristics and results

Author (year) Country	Sample size (N)	Intervention	Study design	Duration (months)	Age (yrs) Female (%)	Initial BMI & Weight (kg)	Endpoint BMI & Weight (kg)	Initial outcome variables	Endpoint outcome variables
Barratt (2008)^17^ England	38	Dietetic led (Lifestyle)	Retrospective case‐control analysis	6	42.9 ± 9.9 100.0	40.49 ± 8.36^a^ 109.53 ± 23.92^a^	37.46^a^ Weight NR	BP^b^: 124/80 HbA1c: 47.2 Cholesterol: 4.80 HDL: 1.30 ± 0.45 LDL: 2.87 ± 0.77 Triacylglycerol: 1.49 ± 0.79	119/79 40.2* 4.79 1.37 ± 0.32 2.81 ± 0.78 1.43 ± 0.97
Brown (2015)^20^ England	828	SWM (SLiM)	Single‐group observational cohort (service evaluation)	6	48.2 ± 11.6 73.7	49.1 ± 9.2 135 ± 28.1	47.6^a^ 131.4^a^	HbA1c: 63.9^a^	59.6^a^^,^ *
Cheyette (2007)^16^ England	49	SWM (Weight No More)	RCT	4	56.7 ± 9.7 47.0	34.1 ± 4.7 97.2 ± 15.1	BMI NR 93.4 ± 14.2	HbA1c: 68.3^a^ Insulin usage: 72.0^a^	65.0^a^ 62.0 ± 30.4
Hughes (2015)^25^ UK ABSTRACT	272	Tier 3	Prospective cohort	12	NR	44.0 123.9	BMI NR 115.6	NR	NR
Jackson (2007)^26^ England	89	Specialist health visitor with expertise in weight management	A prospective before and after study based in one primary healthcare centre	12	55.8 ± 13.8 80.9	37.4 ± 5.85 103.16 ± 16.9	33.11 ± 5.7* 91.64 ± 19.0*	BP: 138.4/78.4 FBS: 5.44 ± 1.08 Cholesterol: 5.38 ± 1.19	124.4/69.6* 5.04 ± 0.60* 5.38 ± 1.33
Jennings (2014)^21^ England	230	Tier 3 SWMS	Single‐group observational cohort (service evaluation)	24	52.7 ± 13.6 70.0	44.1 ± 7.8 124.4 ± 27.3	41.0 ± 7.6* 115.8 ± 26.0	BP: 131/76 Waist: 128 ± 16.2 HbA1c: 57.8 ± 15.3 PA Score: 3.4 ± 1.0	122/71* 118 ± 15.4* 53.7 ± 14.1* 2.8 ± 1.2*
Kininmonth (2016)^22^ Huddersfield, UK ABSTRACT	280	Tier 3 SWMS	Retrospective cohort	6	Age NR 67	49.4 ± 7.4 138.9 ± 27.2	48.5 ± 7.5 136.3 ± 27.5	NR	NR
Lean (2013)^18^ Scotland	91	Low‐energy Liquid diet LELD and Food Reintroduction	Feasibility study	12	45.7 ± 10.7 81.3	48.0 ± 7.6 131.1 ± 25.2	BMI NR 118.7^a^	NR	NR
Logue (2014)^27^ Scotland	1838	Structured educational lifestyle and GCWMS	Prospective observational study	12	49.1 ± 13.5 72.9	43.3 118.1	NR	NR	NR
MacLaughlin (2015)^23^ England	338	Renal Weight Management Programme	Retrospective cohort study	12	52.3 ± 12.8 45.0	36.6 ± 5.3 Weight NR	BMI NR ‐ 4.3 reduction*	NR	NR
McLean (2016)^24^ Scotland	1838	GCWMS for anxiety and depression	Retrospective cohort study	12	48.1 ± 12.5^a^ 72.2^a^	43.77 ± 7.23^a^ 122.5 ± 24.2^a^	NR	NR	NR
Melville (2011)^28^ Scotland	54	(TAKE 5) GCWMS	Before and after study (without control)	6	48.3 ± 12.0 59.3	40.0 ± 8.0 100.6 ± 26.8	39.1 ± 8.2* 96.1 ± 26.9*	Waist: 122.1 ± 15.7	115.8 ± 16.7*
Morrison (2012)^29^ Scotland	2976	SWM GCWMS	Prospective uncontrolled cohort study	24	46.0 72.4	BMI stratified^c^ Weight NR	Stratified	NR	NR
Nield (2016)^30^ England	288	Specialist Community Weight Management Programme	Prospective cohort observational study	6	Age‐stratified 66.0	45.5 ± 6.6 126.9 ± 21.5	43.32^a^^,^ * 120.6^a^^,^ *	PA min/week: 113.2 ± 233.2 Waist: 130.7 ± 14.6	107.4 ± 209.7* 125.0^a^^,^ *
Ross (2008)^31^ England	1906	Counterweight Programme SWM	Prospective uncontrolled cohort study	24	49.4 ± 13.5 77.0	37.1 ± 6.0 101.1	36.02^a^ 98.04^a^	Stratified	Stratified
Rowe (2005)^32^ England	100	Orlistat and behavioural interventions for diet and exercise	Prospective observational without control	24	54.6 ± 11.2^a^ 55.0	39.5 ± 6.5 112.0 ± 20.9^a^	BMI NR 99.7 ± 32.4*	HbA1c: 59.6^a^ Insulin usage: 130 ± 135.4	52.8^a^^,^ * 90 ± 124.1*
Ryan (2017)^33^ England	141	SWMS multidisciplinary, biopsychosocial approach	Before and after study (without control)	12	52.2 ± 11.9 70.0	46.3 ± 7.2 127.2 ± 23.0	BMI NR Weight stratified	Pain: stratified	Stratified
Turner (2015)^15^ Wales, UK	180	MDWMC ‐ Tier 3	Service evaluation by semi‐structured interviews and questionnaires	24	Age NR 72.7^a^	NR	NR	NR	NR
Wright (2012)^19^ Scotland	199	SWMP	Cross‐sectional	6	49.7 ± 12.6 76.4^a^	BMI NR 114.5 ± 23.4	BMI NR 109.4 ± 23.1*	NR	NR

NR, Not Reported.

a Observed, calculated or converted by reviewer.

b Units: BMI (kg/m^2^); Weight (kg); Blood Pressure (BP) (mmHg); HbA1c (mmol/mol); Fasting Blood Sugars (mmol/L); Insulin usage (Units); Cholesterol (mmol/L); HDL& LDL (mmol/L); Triacylglycerol (mmol/L); Waist circumference (Centimetres); Physical Activity (PA) in a) score: where 4 being inactive & 1 active; and in b) minutes per week.

c For more details on stratified data see Appendix Supporting information Tables S4‐S9.

*With statistical significance (ie, *P* < 0.05).

The majority of included studies (95%) reported our primary outcome of interest in weight and/or BMI from the baseline records up to their study endpoint. Turner et al (2015) was the study article that did not report weight in any form at baseline; however, this study reported rates of participants who achieved ≥5% and ≥10% weight reduction at their intervention endpoint of 12 months (ie, 36% and 37%, respectively).^15^

### 3.1 Study design

The study design ranged: one randomized controlled trial (RCT),^16^ a semi‐structured interview (service quality evaluation) study,^15^ a retrospective case‐control,^17^ a feasibility study,^18^ a cross‐sectional,^19^ two single‐group observational cohort (service evaluation) studies,^20^, ^21^ three retrospective (data analysis) cohort studies^22^, ^23^, ^24^ and nine prospective cohort studies.^25^, ^26^, ^27^, ^28^, ^29^, ^30^, ^31^, ^32^, ^33^

Five studies investigated the effect of Tier 3 services.^15^, ^19^, ^21^, ^22^, ^25^ Three looked into the Glasgow and Clyde Weight Management Service (GCWMS).^24^, ^27^, ^29^ Whereas the rest focused on further MWMPs including: “TAKE‐5” GCWMS,^28^ Dietetic led,^17^ “SLiM” SWM,^20^ “Weight No More” SWM,^16^ specialist health visitor programme,^26^ Low‐Energy Liquid Diet (LELD) food reintroduction,^18^ Renal Weight Management Programme (RWMP),^23^ specialist community weight reduction programme,^30^ “CounterWeight” SWM,^31^ Orlistat weight reduction 32 and biopsychological multidisciplinary programme.^33^ Further details on study design and intervention description are in Table 1 and Table S1.

### 3.2 Risk of bias

**Figure 2:**
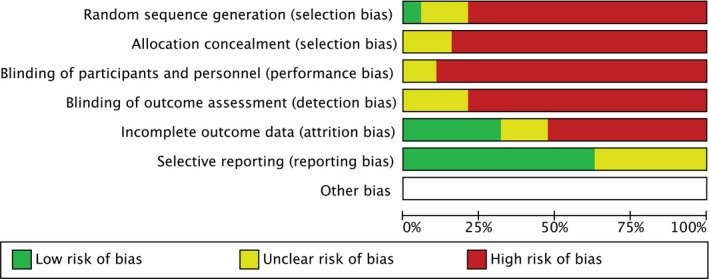
Risk of bias assessment authors' judgements about each risk of bias item for each included study

All studies showed high risk in selection, performance, detection and attrition bias. This is because all included studies, except for the only RCT,^16^ were designed as evaluation (before and after), retrospective analysis or uncontrolled prospective investigation. The risk of publication or reporting bias was low to unclear for all studies which may add to the overall reliability (Figure 2). Attrition bias was evaluated high in consequence of the increased pattern of patients’ drop‐out rate; which was not fully investigated or discussed.

### 3.3 Participants’ characteristics

Women comprised the largest percentage of participants in all except two studies: Cheyette (2007): 47%; and MacLaughlin et al (2015): 45%.^16^, ^23^ One study did not include men (Barratt et al, 2008).^17^ Age ranged from 18 to 75 years; mean age ranged between 40 and 60 years (mean: 49.2 years). Ethnicity was reported in groups by 5 (26%) studies with a clear majority being Caucasian (ranging from 47% to 96%); and with Black African or Asian descendants reported second.^17^, ^20^, ^23^, ^28^, ^30^

### 3.4 Socioeconomic status

SES was reported in 7 (37%) studies in a five‐level scoring classification.^19^, ^24^, ^27^, ^29^, ^30^, ^31^, ^33^ In studies that included SES, the most deprived was reported with the highest rate compared to all other deprivation levels (ranging from 27% to 62%). Only Jennings et al’s (2014) study reported education level, which included three layers (≤15 years: 30%, 15‐19 years: 52%, and ≥19 years: 18%).^21^ In addition, Melville et al’s (2011) study reported participants’ marital status (Married: 2%; Single: 98%) and their type of financial support (Live independently: 7.4%; Family carer: 31.5%; Paid carer: 61.1%).^28^

### 3.5 Primary outcome results

Baseline BMI was reported by 90% of included studies except for Turner et al (2015) and Wright et al (2012) and ranged from 30.1 to 49.1 kg/m^2^.^16^, ^17^, ^18^, ^20^, ^21^, ^22^, ^23^, ^24^, ^25^, ^26^, ^27^, ^28^, ^29^, ^30^, ^31^, ^32^, ^33^ Two studies reported BMI in stratified groupings which left the accumulative average BMI calculated from 16 studies at 42.54 kg/m^2^. Baseline weight in kilograms was also reported by 90% of included studies except for Morrison et al (2011) and Turner et al (2015).^16^, ^17^, ^18^, ^19^, ^20^, ^21^, ^22^, ^23^, ^24^, ^25^, ^26^, ^27^, ^28^, ^30^, ^31^, ^32^, ^33^ Turner et al reported participants that lost weight at 12 months, which was their intervention endpoint.^15^ Wright et al (2012) reported weight at baseline and at six months (114.5 ± 23.4 kg and 109.4 ± 23.1 kg, *P* < 0.001, respectively).^19^ The baseline accumulative average of weight is calculated at 117.88 kg. See Table 2.

**Table 2:** Summary of calculated average primary and secondary outcome results covered and reported by the included studies

	Baseline	3 mo	6 mo	12 mo	18 mo	24 mo
BMI (kg/m^2^)	42.54^(16)^ a	42.40^(6)^	40.73^(8)^	36.67^(3)^		
Weight (kg)	117.88^(16)^	114.48^(7)^	112.17^(10)^	102.89^(5)^	112.0^(1)^	105.95^(2)^
Waist circmf. (cm)	126.9^(3)^	125.3^(2)^	120.3^(3)^	118.0^(1)^		
HbA1c (mmol/mol)	58.8^(5)^	56.5^(2)^	53.8^(5)^	59.4^(2)^		
FBS (mmol/L)	5.44^(1)^	5.08^(1)^	5.14^(1)^	5.04^(1)^		
Insulin usage (Units)	101.0^(2)^	58.7^(1)^	76.55^(2)^	62.0^(1)^		
Cholesterol (mmol/L)	5.09^(2)^	5.18^(1)^	5.01^(2)^	5.38^(1)^		
BP (mmHg)						
Systolic	134.7^(2)^	129.5^(1)^	124.5^(2)^	123.2^(2)^		
Diastolic	77.2	72.6	75.9	70.5		
PA						
Out of 4^b^	3.4	2.9	2.7	2.8		
Min/week^c^	113.2	123.2	107.4			
Drop‐out (%)		9.1^(1)^	33.4^(5)^	44.1^(8)^		74.1^(4)^

a Superscript in‐bracket numbers represent count of studies contributed in calculating the correlated average.

b Inverse score used by Jennings et al (2014) to report physical activity where 4 being inactive and 1 is active.

c Physical activity reported by Nield et al (2016) in minutes per week.

At three months, the calculated average BMI from six studies is 42.40 kg/m^2^; 20, 21, 22, 26, 30, 31 five of which reported statistical significance at (*P* < 0.001).^20^, ^21^, ^26^, ^30^, ^31^ Morrison et al (2011), however, reported BMI in stratification.^29^ The mean reduction in weight ranged from 3.34 ± 3.53 to 4.11 ± 4.95 kg (*P* < 0.001) in 6 studies.^20^, ^21^, ^22^, ^26^, ^30^, ^31^ An average of weight reduction with no BMI informed and with a reported statistical significance by Cheyette (2007) (2.2 kg ±2.7; *P* < 0.01).^16^ In total, eight studies (42%) reported a change in BMI and/or weight at three months from their baseline, and the majority reported statistically significance weight reduction with an accumulative average of 114.48 kg.^16^, ^20^, ^21^, ^22^, ^26^, ^29^, ^30^, ^31^ Six studies (31%) reported a percentage of participants who lost 5% or more of their initial weight (calculated mean: 22.95% of participants).^21^, ^22^, ^24^, ^27^, ^30^, ^31^ Jennings et al (2014) was the only study to report a 10% or more weight reduction rate among participants (3.6%).^21^ Details on rates are summarized in Table 3.

**Table 3:** Calculated average rates of participants who have lost weight covered and reported by the included studies (%)

	3 mo	6 mo	12 mo	18 mo	24 mo
≥5% weight loss	23.98^(7)^ a	39.20^(9)^	43.35^(7)^	47.90^(1)^	44.40^(1)^
≥10% weight loss	3.6^(1)^	10.0^(2)^	29.4^(2)^	26.0^(1)^	20.0^(2)^
≥5 kg weight loss	27.20^(2)^	39.21^(2)^	40.90^(2)^		13.60^(1)^
≥10 kg weight loss			36.0^(1)^		

a Superscript in‐brackets numbers represent count of studies contributed in calculating the correlated average.

At six months, 11 studies (58%) reported changes in BMI or weight (kg) or both.^16^, ^17^, ^19^, ^20^, ^21^, ^22^, ^26^, ^28^, ^30^, ^31^, ^32^ The calculated average reduction in BMI is 1.89 kg/m^2^ ranging from 0.8 to 3.3 kg/m^2^ in eight studies with a cumulative average of 40.73 kg/m^2^.^17^, ^20^, ^21^, ^22^, ^26^, ^28^, ^30^, ^31^ The mean reduction in weight (kg) was reported by ten studies (53%), with a calculated accumulative average of 112.17 kg.^16^, ^19^, ^20^, ^21^, ^22^, ^26^, ^28^, ^30^, ^31^, ^32^ Nine studies (47%) reported a 5% or more weight loss rate among participants with a calculated average of 39.2%.^19^, ^20^, ^21^, ^24^, ^27^, ^28^, ^30^, ^31^, ^32^ Only two studies (11%) reported an average of 10.0% of participants whom lost 10% or more from their initial weight.^20^, ^21^

At one year, five studies (26%) reported a change in BMI or weight or both.^16^, ^21^, ^25^, ^26^, ^31^ BMI reduction was reported by three studies (16%) with a calculated average of 36.67 kg/m^2^.^21^, ^26^, ^31^ Weight reduction was reported by five studies (26%), ranging from 2.8 to 11.6 kg reduction and with a calculated average of 102.89 kg.^16^, ^21^, ^25^, ^26^, ^31^ An average of 43.4% of participants have achieved 5% or more weight loss; as reported by seven studies (37%).^15^, ^21^, ^24^, ^25^, ^27^, ^31^, ^33^ At this point, only two studies (11%) have reported 10% or more weight loss with a calculated average 29.4% of participants.^15^, ^21^

At eighteen months, Jennings et al (2014) was the only study that reported change in kilograms.^21^ The mean reduction in weight was 12.4 kg (*P* < 0.001) with 47.9% of the remaining participants who lost 5% or more and 26% lost 10% or more of their initial weight. At eighteen months, there were no additional outcome variables reported by any of the included studies.

At two years, three studies (16%) briefly reported weight change.^21^, ^29^, ^32^ Jennings et al (2014) and Rowe et al (2005) reported weight change in kilograms from the remaining participants with an average reduction by 11.9 kg (*P* < 0.01) with a cumulated average of 105.95 kg.^21^, ^32^ Morrison et al (2011) reported only the rate of participants that lost 5 kg or more (13.6%).^29^ At this point, there were no additional outcome variables reported by any of the included studies. In addition, no prospective study went beyond two years of follow‐up. Tables 2 and 3 represent calculated average results.

### 3.6 Secondary outcome variables

The included studies reported secondary outcome variables in a heterogeneity that made tracking a set of health outcome variables problematic. Eight studies (42%) reported secondary health outcome variables at baseline: waist circumference, glycaemic control, lipids, BP and physical activity.^16^, ^17^, ^20^, ^21^, ^26^, ^28^, ^30^, ^32^ Details on baseline results are in Table 2.

At three months, Jennings et al (2014) and Nield et al (2016) reported significant reduction in waist circumference by an average of 4.02 cm (*P* < 0.001). The accumulative average of waist circumference was 125.3 cm. They also reported significant increase in physical activity levels, but with different measuring methodology^2^ (Jennings: 17.2%; and Nield: 8.8% increase; *P* < 0.001).^21^, ^30^ Cheyette (2007) and Jennings et al (2014) reported improvements in glycaemic control. The reduction in insulin usage reported by Cheyette is 10.1 ± 16.4 units (*P* < 0.01); and an average of 56.5 mmol/mol in HbA1c^3^ reported by two studies.^16^, ^21^ Jackson et al (2007) reported a significant improvement in FBS by a reduction by 0.36 mmol/L from baseline. Jackson also reported improvement in BP with a significant mean reduction of 9.0 mmHg systolic and 5.8 mmHg diastolic (*P* < 0.001) and a mean reduction in cholesterol by 0.2 mmol/L (*P* = 0.02; Table 2).^26^

At six months, three studies (16%) reported further significant reduction in waist circumference with an average of 6.6 cm (*P* < 0.001).^21^, ^28^, ^30^ The waist circumference averaged at 120.3 cm. The average reduction in HbA1c from five studies (26%) is calculated at 4.86 mmol/mol (*P* < 0.05).^16^, ^17^, ^20^, ^21^, ^32^ Rowe et al (2005) reported further significant reductions in insulin usage by a calculated mean of 40.0 units (*P* < 0.001).^32^ In addition, Jackson et al (2007) indicated a constant decrease in FBS by 0.3 mmol/L from baseline (*P* = 0.03).^26^ Jackson also reported an insignificant reduction in cholesterol (by 0.15 mmol/L; *P* = 0.6). Jennings et al (2014) reported increase in physical activity (by 26%; *P* < 0.001) from baseline; whereas Nield et al (2016), reported a decline (from 123.2 min/wk at 3 months to 107.4 min/wk at 6 months).^21^, ^30^ The calculated average reduction in BP was reported by two studies (11%); with an average reduction in systolic BP by 10.2 mmHg and diastolic by 1.3 mmHg from baseline.^21^, ^30^ Five studies reported the drop‐out rate with an average of 33.4%, ranging from 18% to 60% (Table 2).^20^, ^22^, ^28^, ^30^, ^32^

At one year, HbA1c average results calculated from two studies (11%) was found to reclaim to the baseline calculated average (59.4 compared to 58.8 at baseline).^16^, ^21^ Turner et al (2015), however, noted that 36% of participants reported a reduction in insulin usage.^15^ Cheyette's (2007) participants experienced a similar reduced level of mean insulin usage as they did at three months (62.0 ± 30.4 units).^16^ Similarly, Jackson et al’s (2007) participants had FBS tested as similar levels as three months of intervention (5.04 ± 0.60 mmol/L). Jackson also reported an insignificant change in cholesterol.^26^ Both Jackson et al (2007) and Jennings et al (2014) reported a statistically significant decrease in BP with an average systolic reduction of 11.5 mmHg and in diastolic by 6.76 mmHg (*P* = 0.001).^21^, ^26^ Only one study (6%) reported physical activity with a similar level as the three‐month point of intervention (scored 2.8 compared to 2.9 at three months).^21^ Waist circumference remained relatively constant compared to six‐months point; with a mean reported by one study 118.8 cm.^21^ Eight studies reported increased drop‐out rate with an average of 44.1% ranging from 15.6% to 78.3% (Table 2).^18^, ^21^, ^23^, ^25^, ^26^, ^27^, ^31^, ^33^

At eighteen and twenty‐four months, there were little or no secondary outcome variables reported by any of the included studies. Drop‐out rate increased to an average of 74.13% at two years point; ranging from 62.0% to 80.5%, as reported by 4 studies.^21^, ^29^, ^31^, ^32^ Table 2 summarize drop‐out rates form included studies.

## 4 DISCUSSION

Although obesity has an increasing acadaemic and clinical interest globally, the evidence on Tier 3 and all other MWMPs in the UK remains scarce.^4^ The aim of the present review was to examine Tier 3 and MWMPs for severely obese adults. Our review supports the accumulating available evidence that Tier 3 intervention reached positive influence on morbidly and among severely obese patients in the pre‐bariatric stage. Evidence suggests that Tier 3 interventions are effective obesity treatment, especially during the early months of involvement.

In general, all MWMPs were found to reduce weight considerably and to improve other health outcomes measured from baseline on most reported variables. The magnitude of the effect, however, seems to lose momentum after six months of intervention. This later observation is crucial with regards to the appropriate timing for a bariatric surgical intervention. A small number of included studies discuss this phenomenon, perhaps due to the substantial proportion of participants who drop‐out at an accelerating rate beyond the three‐month point of intervention. In addition, more recent studies have provided novel insights into the processes and mechanisms that underpin weight regain after weight loss. In addition to environmental and behavioural factors, physiological (or metabolic) adaptations to weight loss favour weight regain due to perturbations in the levels of circulating appetite‐related hormones and energy homoeostasis, in addition to alterations in nutrient metabolism and subjective appetite. To maintain weight loss, individuals must adhere to behaviours that counteract physiological adaptations and other factors favouring weight regain.^34^, ^35^ It is difficult to overcome physiology with behaviour. Nonetheless, this, and variations in study duration may contribute to preventing this review from comparing the true effect size between included studies. Though future research is required to examine secondary outcome variables such as glycaemic control and lipids (in stratifications) extensively, weight loss goals such as 5% weight loss (NICE guidelines) are reachable at early stages of interventions (Table 3).

We agree with Brown et al’s (2017) review, which notes most available reviewed evidence comes from observational studies in which randomized selection and allocation into Tier 3 services would improve inference reliability.^12^ The only RCT reviewed, for instance, lasted for a short intervention duration (four months) and reported a modest mean reduction in weight (2.2 kg).^16^ At three months, the mean reduction in weight from all studies that reported changes (including the RCT) reached 4.11 kg, thus almost doubling the reported RCT‐measured effect.

Improvements in secondary health outcome variables are significant until the effects of the drop‐out rate become apparent. This may be because all studies have excluded drop‐out data from their analyses at each interval. At the three‐ and six‐months points, however, we can appreciate achieved improvements in glycaemic control and BP. Most studies that reported secondary outcome variables related magnitude to a statistical significance in physical activity, for instance, the average increase reached 26% at three months (*P* < 0.001) but declined afterwards.^21^, ^30^ Despite the high risk of bias assessment, we have noticed no difference in magnitude between small and large sample size studies. Studies that reported demographic characteristics such as SES and/or education levels did not reveal distinct effects either. Thus, Tier 3 and MWMPs may have been preventive tools in the short‐ and mid‐term, treating obesity regardless of sample size, demographic characteristics and/or comorbidities.

About the interpretation of data, we noted that studies invested in patients’ emotional and motivational status, and which reported data for depression and anxiety, were just as likely to have a high rate of patient drop‐outs as those that did not. This, in count, does not support the notion that weight reduction levels in those programmes were superior to other studies that did not target emotional health. McLean et al (2016), for instance, concluded that patients with complex obesity who scored high for severe anxiety and/or depression participating in an MWMP with integrated psychological support, achieved similar weight reduction outcomes compared to non‐severe cases.^24^ Thus, more research is needed regarding obese people's mental wellbeing, process and pathway for psychological intervention as well as robust outcomes from such interventions.

A majority of included studies were not as precise in discussing participants’ reasons for dropping out. Extending efforts to assess and overcome drop‐outs appeared to contribute to a successful intervention (especially a multicomponent one) and the achievement of desired targets. This is because, as anticipated by commissioning parties, Tier 3’s main goal is to help patients, at a minimum, to lose weight and improve most of their quality of life aspects, improve and induce remission of comorbidities or to optimize patients’ preparation for a Tier 4 bariatric surgical intervention. The goal is, optimistically, helping patients to take control of their own lives and all other healthful elements; which is the drive for commissioning all tiered weight reduction interventions.

Brown et al (2017) recently published a systematic review examining a set of criteria for interventions similar to the ones this review has covered.^12^ We have only excluded two studies from their selection, as one was of non‐British origin and the other was comparing groups in post‐bariatric.^13^, ^36^ They reviewed 14 studies, and our conclusions were based on lines of theoretical analysis similar to theirs. Our review adds to the evidence base on a stratified basis with summaries for weight loss achieved and calculated average outcome results and suggests further research regarding intervention's high drop‐out rates as well as outcomes from psychological and physical activity interventions. More RCT‐designed studies would greatly contribute to robust, real‐life findings, as all possible confounding effects would ideally distribute evenly.

## 5 LIMITATIONS

Studies published on Tier 3 and UK MWMPs are limited in number. Yet, most if not all of included studies are of high risk of bias in terms of allocation sequence, allocation concealment, blinding, incomplete outcome data. The only RCT reviewed has shown a modest change in weight compared to all included studies.^16^ The high rate of drop‐outs was present in most if not all included studies with inadequate reasoning. The majority have excluded non‐completers’ data from their final analysis.

## 6 CONCLUSION

The reviewed evidence for the Tier 3 service and MWMPs suggests a short‐ to mid‐ranged positive effect on British patients with obesity (BMI ≥30 kg/m^2^) regarding accumulated reduction in weight, glycaemic control, BP and subtle improvement in physical activity. The high drop‐out rate might have contributed to limiting longer terms’ progress in all positive results, especially those related to physical activity. More randomized trial investigations and drop‐out explorations would improve overall reliability. Tier 3 service and MWMPs can assist obese adults living in the UK to lose weight and may improve their overall health status.

## ETHICS STATEMENT

Since this is a systematic review, ethical request is not applicable.

## CONFLICT OF INTEREST

No conflict of interest is declared for all authors.

## AUTHORS CONTRIBUTION

Alkharaiji, M undertook data (study) collection, analysis and wrote the first draft of the manuscript. Anyanwagu, U acted as an independent second reviewer for study selection, supported analysis and supported the final draft of the manuscript. Donnelly, R. provided crucial academic input on the content of the manuscript and interpretation of data analysis. Idris, I. conceived the study, provided academic supervision, supported data analysis and interpretation, and wrote the final draft of the manuscript.

## Data Availability

Supporting data are provided as supplementary information.

## ENDNOTES

1 Reported and accumulated HbA1c average results in this review will be converted to mmol/mol.

2 Jennings et al (2014) reported physical activity through a 4‐level scoring methodology with score number 4 being inactive and score number 1 being active. Nield et al (2016) used minutes per week as the unit of measurement

3 Calculated average after conversion from percent to mmol/mol measurement units.

## References

[1] Welbourn R , Dixon J , Barth JH , et al. NICE‐Accredited commissioning guidance for weight assessment and management clinics: A model for a specialist multidisciplinary team approach for people with severe obesity. Obes Surg. 2016;26(3):649‐659. 26738895 10.1007/s11695-015-2041-8

[2] Clinical commissioning policy . Complex and Specialised Obesity Surgery. UK: NHS Commissioning Board; 2013 https://www.england.nhs.uk/wp-content/uploads/2016/05/appndx-6-policy-sev-comp-obesity-pdf.pdf. Accessed September 01, 2018.

[3] Health and social care information centre . Statistics on Obesity: Physical Activity and Diet. UK: NHS; 2016 https://files.digital.nhs.uk/publicationimport/pub20xxx/pub20562/obes-phys-acti-diet-eng-2016-rep.pdf. Accessed September 01, 2018.

[4] British Obesity and Metabolic Surgery Society . Commissioning Guide: Weight Assessment and Management Clinics (Tier 3). London, UK: British Obesity and Metabolic Surgery Society; 2014.

[5] Dobbs R , Sawers C , Thompson F , et al. Overcoming Obesity: An Initial Economic Analysis. London, UK: McKinsey Global Institute; 2014.

[6] Buchwald H , Oien DM . Metabolic/bariatric surgery worldwide 2011. Obes Surg. 2013;23(4):427‐436. 23338049 10.1007/s11695-012-0864-0

[7] Ryan DH . New medications and new ways to use them. Obesity. 2015;23(Suppl 1):S1‐S4. 10.1002/oby.21091 25900866

[8] Welbourn R , le Roux CW , Owen‐Smith A , Wordsworth S , Blazeby JM . Why the NHS should do more bariatric surgery; how much should we do? BMJ. 2016;353:1‐5. 10.1136/bmj.i1472 27169605

[9] DeVille‐Almond J . Overweight and Obese Adults: Lifestyle Weight Management, in Nursing Times (pp. 15). London: Emap Limited; 2014. 25137946

[10] Anderson B , et al. Obesity: Clinical Assessment and Management [Quality Standard]. UK: National Institute for Health and Care Excellence; 2016 https://www.nice.org.uk/guidance/qs127/resources/obesity-clinical-assessment-and-management-pdf-75545363615173. Accessed Accessed September 01, 2018.

[11] Stegenga H , Haines A , Jones K , Wilding J . Identification, assessment, and management of overweight and obesity: Summary of updated NICE guidance. BMJ. 2014;349:g6608‐g6608. 25430558 10.1136/bmj.g6608

[12] Brown TJ , O'Malley C , Blackshaw J et al. Exploring the evidence base for Tier 3 weight management interventions for adults: a systematic review. Clin Obes. 2017;7(5):260‐272. 28695579 10.1111/cob.12204

[13] Patel P , Hartland A , Hollis A et al. Tier 3 multidisciplinary medical weight management improves outcome of Roux‐en‐Y gastric bypass surgery. Ann R Coll Surg Engl. 2015;97(3):235‐237. 26263811 10.1308/003588414X14055925061838 PMC4474019

[14] Higgins J , Green S . Cochrane Handbook for Systematic Reviews of Interventions [Version 5.1.0]. The Cochrane Collaboration; 2011 http://handbook-5-1.cochrane.org. Accessed September 01, 2018.

[15] Turner D , Haboubi N . Qualitative and quantitative outcomes of a 1:1 multidisciplinary weight management clinic. Healthcare. 2015;3(2):429‐451. 27417772 10.3390/healthcare3020429 PMC4939542

[16] Cheyette C . Weight no more: A randomised controlled trial for people with type 2 diabetes on insulin therapy. Pract Diabet Int. 2007;24(9):450‐456.

[17] Barratt R , Frost G , O’Boyle A , Millward J , Truby H . Use of sibutramine to assist obese women with weight loss can be successful in dietitian‐led clinics: another tool in the dietitian’s toolbox. J Hum Nut Diet. 2008;21(3):248‐255. 10.1111/j.1365-277X.2008.00870.x 18477180

[18] Lean M , Brosnahan N , McLoone P et al. Feasibility and indicative results from a 12‐month low‐energy liquid diet treatment and maintenance programme for severe obesity. Br J Gen Pract. 2013;63(607):e115. 23561690 10.3399/bjgp13X663073 PMC3553637

[19] Wright F , Boyle S , Baxter K et al. Understanding the relationship between weight loss, emotional well‐being and health‐related quality of life in patients attending a specialist obesity weight management service. J Health Psychol. 2012;18(4):574‐586. 22843633 10.1177/1359105312451865

[20] Brown A , Gouldstone A , Fox E et al. Description and preliminary results from a structured specialist behavioural weight management group intervention: Specialist Lifestyle Management (SLiM) programme. BMJ open. 2015;5(4):e007217. 10.1136/bmjopen-2014-007217 PMC4390730 25854970

[21] Jennings A , Hughes CA , Kumaravel B et al. Evaluation of a multidisciplinary Tier 3 weight management service for adults with morbid obesity, or obesity and comorbidities, based in primary care. Clin Obes. 2014;4(5):254‐266. 25825858 10.1111/cob.12066 PMC4253319

[22] Kininmonth A , Bradbury J . Evaluation of a Tier 3 specialist weight management service for morbidly obese patients. Proc Nutr Soc. 2016;75(3):E203.

[23] MacLaughlin HL , Hall WL , Condry J , Sanders T , Macdougall IC . Participation in a structured weight loss program and all‐cause mortality and cardiovascular morbidity in obese patients with chronic kidney disease. J Ren Nut. 2015;25(6):472‐479. 10.1053/j.jrn.2015.05.001 26143293

[24] McLean RC , Morrison DS , Shearer R , Boyle S , Logue J . Attrition and weight loss outcomes for patients with complex obesity, anxiety and depression attending a weight management programme with targeted psychological treatment. Clin Obes. 2016;6(2):133‐142. 26842226 10.1111/cob.12136

[25] Hughes C , Steel N . A service evaluation of a primary care Tier 3 weight management service using the National Obesity Observatory Standard Evaluation Framework. Appetite. 2015;87:377‐377.

[26] Jackson C , Coe A , Cheater FM , Wroe S . Specialist health visitor‐led weight management intervention in primary care: exploratory evaluation. J Adv Nurs. 2007;58(1):23‐34. 17394613 10.1111/j.1365-2648.2007.04226.x

[27] Logue J , Allardice G , Gillies M , Forde L , Morrison DS . Outcomes of a specialist weight management programme in the UK National Health Service: Prospective study of 1838 patients. BMJ open. 2014;4(1):e003747. 10.1136/bmjopen-2013-003747 PMC3902487 24394799

[28] Melville CA , Boyle S , Miller S et al. An open study of the effectiveness of a multi‐component weight‐loss intervention for adults with intellectual disabilities and obesity. Br J Nutr. 2011;105(10):1553‐1562. 21255473 10.1017/S0007114510005362

[29] Morrison DS , Boyle S , Morrison C , Allardice G , Greenlaw N , Forde L . Evaluation of the first phase of a specialist weight management programme in the UK National Health Service: Prospective cohort study. Public Health Nutr. 2011;15(1):28‐38. 21806868 10.1017/S1368980011001625

[30] Nield L , Kelly S . Outcomes of a community‐based weight management programme for morbidly obese populations. J Hum Nutr Diet. 2016;29(6):669‐676. 27357098 10.1111/jhn.12392

[31] Ross HM , Laws R , Reckless J , Lean M . Evaluation of the Counterweight Programme for obesity management in primary care: a starting point for continuous improvement. Br J Gen Pract. 2008;58(553):548–548. 18682018 10.3399/bjgp08X319710 PMC2486382

[32] Rowe R , Cowx M , Poole C , McEwan P , Morgan C , Walker M . The effects of orlistat in patients with diabetes: improvement in glycaemic control and weight loss. Curr Med Res Opin. 2005;21(11):1885–1890. 16307710 10.1185/030079905X74943

[33] Ryan CG , Vijayaraman A , Denny V , et al. The association between baseline persistent pain and weight change in patients attending a specialist weight management service. PloS one. 2017;12(6):e0179227. 28604789 10.1371/journal.pone.0179227 PMC5467875

[34] Hall KD , Hammond RA , Rahmandad H . Dynamic interplay among homeostatic, hedonic, and cognitive feedback circuits regulating body weight. Am J Public Health. 2014;104(7):1169–1175. 24832422 10.2105/AJPH.2014.301931 PMC4056226

[35] Greenway FL . Physiological adaptations to weight loss and factors favouring weight regain. Int J Obes. 2015;39(8):1188–1196. 10.1038/ijo.2015.59 PMC4766925 25896063

[36] Crowe C , Gibson I , Cunningham K , et al. Effects of an eight‐week supervised, structured lifestyle modification programme on anthropometric, metabolic and cardiovascular risk factors in severely obese adults. BMC Endocr Disord. 2015;15(1):37–37. 26231181 10.1186/s12902-015-0038-x PMC4522055

[37] Moher D , Liberati A , Tetzlaff J , Altman DG . Preferred reporting items for systematic reviews and meta‐analyses: The PRISMA Statement. PLOS Med. 2009;6(7):e1000097. 19621072 10.1371/journal.pmed.1000097 PMC2707599

